# Detection and Genome Sequence Analysis of Avian Metapneumovirus Subtype A Viruses Circulating in Commercial Chicken Flocks in Mexico

**DOI:** 10.3390/vetsci9100579

**Published:** 2022-10-19

**Authors:** Henry M. Kariithi, Nancy Christy, Eduardo L. Decanini, Stéphane Lemiere, Jeremy D. Volkening, Claudio L. Afonso, David L. Suarez

**Affiliations:** 1 Exotic and Emerging Avian Viral Diseases Research Unit, Southeast Poultry Research Laboratory, U.S. National Poultry Research Center, USDA-ARS, Athens, GA 30605, USA; 2 Biotechnology Research Institute, Kenya Agricultural and Livestock Research Organization, Kaptagat Rd, Nairobi P.O. Box 57811-00200, Kenya; 3 Boehringer Ingelheim Animal Health, Guadalajara 44940, Mexico; 4 Boehringer Ingelheim Animal Health IMETA, Dubai P.O. Box 507066, United Arab Emirates; 5 Boehringer Ingelheim - Gerland, 69007 Lyon, France; 6 BASE2BIO, Oshkosh, WI 54904, USA

**Keywords:** aMPV, cluster, de novo assembly, FTA card, NGS, SEP rRT-PCR, *Pneumoviridae*

## Abstract

**Simple Summary:**

The use of next-generation sequencing to identify pathogens in clinical samples is continually improving, providing genomic data that can be used to: (1) identify what viruses are present in particular samples, (2) determine the viral genotype, pathotype, or lineage, (3) predict which viruses are likely to cause severe disease, and (4) help guide what vaccines to use for control efforts. As part of a project to improve NGS for diagnostics, samples were collected from commercial chicken farms in Mexico and transported on FTA cards. One of the commonly identified viruses was avian metapneumovirus, an important upper respiratory avian pathogen consisting of subtypes A-D. Complete genomes of seven subtype-A isolates were identified, which are distinct from other previously reported subtype-A strains. Although subtype-A viruses were previously reported in Mexico, this is the first comprehensive complete genome sequence analysis of Mexican subtype-A viruses. These data provide a much clearer picture of viruses circulating in Mexico and demonstrate the value of next-generation sequencing to identify pathogens where surveillance is not routinely performed. Because subtype-A is not found in the U.S., a modified real-time reverse-transcriptase polymerase chain reaction test was evaluated that can quickly identify the virus in poultry samples.

**Abstract:**

Avian metapneumoviruses (aMPV subtypes A-D) are respiratory and reproductive pathogens of poultry. Since aMPV-A was initially reported in Mexico in 2014, there have been no additional reports of its detection in the country. Using nontargeted next-generation sequencing (NGS) of FTA card-spotted respiratory samples from commercial chickens in Mexico, seven full genome sequences of aMPV-A (lengths of 13,288–13,381 nucleotides) were de novo assembled. Additionally, complete coding sequences of genes N (*n* = 2), P and M (*n* = 7 each), F and L (*n* = 1 each), M2 (*n* = 6), SH (*n* = 5) and G (*n* = 2) were reference-based assembled from another seven samples. The Mexican isolates phylogenetically group with, but in a distinct clade separate from, other aMPV-A strains. The genome and G-gene nt sequences of the Mexican aMPVs are closest to strain UK/8544/06 (97.22–97.47% and 95.07–95.83%, respectively). Various amino acid variations distinguish the Mexican isolates from each other, and other aMPV-A strains, most of which are in the G (*n* = 38), F (*n* = 12), and L (*n* = 19) proteins. Using our sequence data and publicly available aMPV-A data, we revised a previously published rRT-PCR test, which resulted in different cycling and amplification conditions for aMPV-A to make it more compatible with other commonly used rRT-PCR diagnostic cycling conditions. This is the first comprehensive sequence analysis of aMPVs in Mexico and demonstrates the value of nontargeted NGS to identify pathogens where targeted virus surveillance is likely not routinely performed.

## 1. Introduction

Four subtypes of avian metapneumoviruses (aMPV-A to D; *Pneumoviridae* family) are currently recognized, based on sequence variations of the surface attachment (G) gene and antigenic differences between strains. Two recently discovered viruses in a parrot and a gull in North America are related to avian metapneumovirus species and are putative new subtypes [1,2,3,4,5,6]. Subtypes A, B, and C have been detected primarily in chickens and turkeys, but adaptation to unconventional hosts has been suggested [7]. The aMPVs are associated with acute and highly contagious upper respiratory tract infections and reproductive problems in turkeys, chickens, and ducks, which can be worsened by coinfections with other viral and secondary opportunistic bacterial pathogens [8,9]. Diagnosis of aMPV infection is difficult because the clinical signs consistent with respiratory disease are not specific to the virus. The virus is primarily detected by serological (ELISA) and molecular (conventional or real-time reverse transcriptase polymerase chain reaction (PCR/rRT-PCR) techniques [10,11,12]. However, sequence variation in aMPV subtypes necessitated the development of subtype-specific rRT-PCR tests. Direct next-generation sequencing (NGS) of cDNAs from total RNA extracts of field-collected samples is a promising diagnostic test, but because of its higher cost, longer processing time, and bioinformatics expertise required for data analyses, rRT-PCR remains the primary front-line test.

The negative-sense, unsegmented linear RNA genome of aMPV (~ 13.3–14 kb in size) contains eight genes encoding nine proteins: nucleocapsid protein (N), phosphoprotein (P), matrix (M), fusion (F), accessory matrix glycoprotein (M2, with two overlapping M2-1 and M2-2 proteins), small hydrophobic (SH), attachment (G) and RNA-dependent RNA polymerase (L), which are organized as 3′-leader-N-P-M-F-M2-SH-G-L-trailer-5′ [13,14]. The viral envelope is embedded with F (promotes viral-to-cell membrane fusion), G (a major antigenic determinant involved in viral attachment), and SH (a viroporin involved in membrane permeability) proteins [13,14,15]. The G protein of aMPV-A is thought to be dispensable to viral viability in cell culture and infected turkeys [16,17], but in aMPV-C, the protein is associated with virus–host immune interactions [18,19]. Proteins N, P, and L encapsulate the viral RNA to form the viral ribonucleoprotein complex (RNP), while the accessory proteins M2-2 and M2-1 are involved in viral genome replication and the 3′-leader/5′-trailer contain viral transcriptional promoters [13,14,20].

The aMPV infections are managed by implementing strict hygiene and biosecurity measures (to limit virus spread and increase vaccine efficacy) and using live-attenuated, killed and recombinant vaccines [21]. When correctly administered, these vaccines elicit effective immune responses, but they ineffectively block respiratory infections (e.g., when using killed vaccines) and viral transmission, which could account for the frequent report of aMPV-associated disease outbreaks in vaccinated flocks [22,23,24,25]. Further, the reversion to virulence of live vaccine strains and the emergence of novel field variants capable of avoiding vaccine-induced immunity compromise vaccination efforts and potentially contribute to persistent viral infections and disease outbreaks [23,26,27,28].

The aMPV was initially detected in turkeys exhibiting respiratory disease in South Africa in 1978 and, subsequently, detected in Europe, Israel, Asia, and Africa [21,29,30,31,32]. The virus is currently globally distributed (except in Australia), and is presently considered among the major respiratory pathogens in commercial poultry-producing regions [13]. Long-distance migratory birds are implicated in the intercontinental spread of the virus, but it is unclear to what extent they contribute to virus spread in domestic poultry flocks [21,33,34]. The aMPV-A and -B viruses are widespread globally (except in North America and Australia). aMPV-C (consisting of North American and Eurasian lineages) was endemic in turkeys in the North Central USA before a successful eradication program, but it is likely endemic in some wild avian species; aMPV-C has recently been reported in wild mallards in the Netherlands and Italy [35,36]. Sporadic detections of aMPV-C in some avian species of minor economic importance (e.g., Muscovy duck and pheasant) in South Korea, China, and France, and aMPV-D in farmed turkey flocks in France, have also been reported [2,21,37,38,39,40,41,42,43]. In South America, aMPV-A and B viruses are predominantly detected in commercial chicken flocks and wild birds in Brazil. In Mexico, aMPV-A viruses were initially reported in 2014 in some poultry farming zones [44,45,46,47]. However, most of the aMPV genomic data available in GenBank are partial gene sequences detected using conventional molecular detection assays.

Here, we report the molecular characterization of aMPVs identified using direct random NGS of RNA from FTA card-spotted clinical samples collected from commercial chicken flocks from Mexico. Further, using bioinformatics, we reviewed the publicly-available aMPV sequence data and the additional data from this paper to modify and optimize a previously published rRT-PCR test for improved specificity and sensitivity to aMPV-A viruses in poultry [10,11].

## 2. Materials and Methods

### 2.1. Samples

We analyzed 265 samples from commercial broiler (*n* = 251) and layer (*n* = 14) chicken flocks from Mexico between May 2019 and April 2022. Metadata on the health (e.g., observable clinical signs consistent with respiratory disease) and vaccination histories of the sampled flocks was not provided. The samples were from respiratory (choana, lung, and trachea; *n* = 149), immunological (spleen and bursa; *n* = 114) and digestive (cloaca; *n* = 2) tissues swabbed using standard procedures from 100 birds per flock. Then, 25 samples from each flock were pooled (i.e., 25 × 4 = 100 samples) in sterile 1.5 mL viral transport media, and each of the four pools was spotted on a 4-sample-area (125 µL of one pool per area) Whatman FTA cards^®^ (Millipore-Sigma, St. Louis, MO, USA). Each sample-spotted FTA card was treated as a single sample for subsequent analysis and shipped to the Southeast Poultry Research Laboratory (SEPRL), USDA-ARS, Athens, GA, for storage (−80 °C) in a BSL-3E laboratory until further processing.

### 2.2. RNA Extraction

Twenty-four 3-mm discs (i.e., six discs per sample-spotted area) were punched out from each sample-spotted FTA card using disposable punches (Robbins Instruments, Houston, TX, USA) and incubated (30 min at RT) in 240 µL of nuclease-free TE buffer (10 mM Tris-HCl; 0.1 mM EDTA, pH 8.0) to dissolve nucleic acids. Total RNA was extracted from 150 µL of the TE/sample eluate using MagMAX™-96 AI/ND Viral RNA Isolation Kit (Thermo Fisher Scientific, Waltham, MA, USA), eluted in 50 µL of elution buffer, and 12 µL subsequently treated with our recently published in-house RNaseH rRNA depletion protocol [48] to selectively deplete host-specific rRNAs (18S, 28S and mitochondrial) and selected bacterial rRNAs (16S/23S) prior to preparation of sequencing libraries.

### 2.3. Library Preparation and NGS

Sequence-independent, single-primer amplification (SISPA) [49] was used to synthesize cDNAs from 10 µL of the RNaseH-treated RNA using random K-8N primer with SuperScript ^TM^ IV First Strand Synthesis Kit (Invitrogen, Waltham, MA, USA) and Klenow polymerase (NEB Inc., Ipswich, MA, USA) kits. After bead-purification (Agencourt AMPure XP Kit; Beckman Coulter Life Sciences, Indianapolis, IN, USA), 5 µL of the cDNAs were amplified using Phusion^®^ High-Fidelity PCR Kit (NEB Inc., Ipswich, MA, USA) and used to prepare sequencing libraries using Nextera ^TM^ DNA Flex kit (Illumina, San Diego, CA, USA) following manufacturer’s recommendations. Based on their concentrations and average fragment sizes as determined using Qubit™ dsDNA HS Assay Kit (Thermo-Fisher Scientific, Waltham, MA, USA) and Agilent 4150 TapeStation HS D5000 System (Agilent Technologies, Inc., Santa Clara, CA), respectively, the libraries were pooled (4 nM, 8 µL each) and then digested with 0.2 N NaOH (5 min incubation at RT). After the addition of a control library (5% PhiX library v3) to the diluted library pools (10 pM final concentration), paired-end sequencing (2 × 300 bp) was performed using the 600-cycle MiSeq Reagent Kit v3 (Illumina, San Diego, CA, USA) on an Illumina MiSeq instrument. Each MiSeq run consisted of 48 samples.

### 2.4. Sequence Assembly, Annotation, and Comparative Genomics

Raw NGS data were processed using a nontargeted classification and assembly pipeline developed by BASE₂BIO LLC (Oshkosh, WI, USA). Briefly, a Nextera adaptor and SISPA primer sequences were removed using TrimGalore v0.6.7 [50]. Taxonomic classification was performed with KrakenUniq v0.5.8 [51], modified with local patches, using in-house hierarchical *k-mer* databases compiled from vector/contaminants, and host (*Gallus gallus*), human, reference bacterial, fungal, archaeal, and protist genomes, and all viral genomes available from GenBank. Read classifications were further filtered using a patched version of the ‘krakenuniq-filter’ package script, requiring a minimum taxon-specific *k-mer* fraction of 0.05 for viral taxa and 0.25 for all other taxa. Individual taxonomic identifications were further verified using BLASTn search [52] of *k-mer* classified reads against the GenBank BLAST ‘nt’ database and subsequent lowest common ancestor (LCA) assignment using in-house software.

For aMPV-positive samples, taxon-specific trimmed reads were extracted based on KrakenUniq classification tables, which were used as input for genome assembly. For high-coverage samples, reads were assembled with both MEGAHIT v1.2.9 [53] and SPAdes v3.14.1 (default settings), with the optimal assembly chosen based on contiguity and comparative alignment to a reference sequence. For low-coverage samples, reference-based consensus calling was performed. Reads were mapped against the reference sequence turkey/UK/8544/06 (GenBank accession number DQ666911) using BWA-MEM v0.717-r1188 [54]. Consensus sequence calling (minimum base quality of 10; minimum read depth of 2×) was performed using the ‘bam2consensus’ program v0.004 [55]. Regions of missing or insufficient read depth were filled with poly-N. For lower-coverage regions where short indels were detected that would introduce frameshifts in the core viral genes, and where examination of the read alignments showed reads supporting both in-frame and frameshift variants, the in-frame variant was used in the final consensus sequences. Viral termini were cleaned and trimmed using the ‘virus_term_polish’ tool [55], which attempts to extend or trim assembly ends to match the reference genome terminal sequences supported by raw read data.

Geneious Prime^®^ v2022.2.0 software (www.geneious.com) was used to predict open reading frames (ORFs; minimum size set at 50 nucleotides (nt) from start to stop codons), to annotate corresponding genes, and to compare with homologous genes/ORFs and coding sequences (CDS) of aMPVs retrieved from GenBank. Protein domain analyses were performed using Geneious Prime, and potential glycosylation sites were predicted using NetNGlyc v1.0 and NetOGlyc v4.0 suites [56,57].

### 2.5. Phylogenetics

Genome and specific gene sequences obtained from this study, together with sequences of representative aMPV subtypes A-D (retrieved from GenBank) were used for multiple sequence alignment using MAFFT v7.490 [58] executed in Geneious Prime^®^. The aligned sequences were trimmed using trimAl tool v1.3 [59], followed by phylogenetic analysis using the maximum likelihood method in MEGA with 1000 bootstrap replicates of the original data and the best model automatically identified by MEGA; all positions with less than 95% site coverage were eliminated from phylogenetic tree reconstructions [60].

### 2.6. aMPV-Specific rRT-PCR Test

#### 2.6.1. Bioinformatics

Two published rRT-PCR tests based on aMPV’s N- and G-genes [10,11] were considered for evaluation and optimization into a sensitive and specific test for aMPV-A. Since fewer N-gene sequences are available in GenBank, G-gene sequences (*n* = 45 from GenBank and seven from this study) were used for single nucleotide polymorphism (SNP) analysis executed in the Virus Pathogen Database and Analysis Resource [61]. The average SNP scores (zero to 200, fully conserved and completely random, respectively) were calculated for the entire primer and probe as well as regions upstream and downstream where a rolling boxcar average was calculated for 20 nt segments to evaluate the conservation of the G-gene primer and probe sequences to the available isolates. This analysis can identify the most conserved regions of the aMPV genome, but additional factors for design are also considered to evaluate existing or new primers and probes (Tm of oligonucleotides, GC percentage, location of mismatches in primer, etc.). From this analysis, the following alternative, more conserved reverse sequence primer and probe, which overlapped with the original primer and probe [10], were developed: AmPV-A + primer (5′-GGA CAT CGG GAG GAG GTA CA -3′), AmPV-A – SEP primer (5′- CTG CAC TCC TCT AAC ACT GAC TGT T -3′) and AmPV-A SEP probe (5′- FAM – CTG ACC TGC ACA GTC ACT ATT GCA CTC ACT GT – BHQ1 -3′).

#### 2.6.2. rRT-PCR Testing

The above-mentioned (new and original) primers and probes [10] were compared at both the original cycling conditions and the three-step cycling program used in other rRT-PCR tests that allow for multiple-test runs on the same 96-well plate. For compatibility with the reagents that are commonly used in the U.S. veterinary diagnostic laboratories, the reagents of the QuantiTect Probe RT-PCR kit (Qiagen, USA) described in the original paper [10] were substituted with the AgPath-ID™ One-Step RT-PCR Reagents (Thermo Fisher Scientific). Ten-fold diluted RNAs were used to determine the detection endpoint on the SmartCycler RT-PCR machine. The SEP aMPV-A reaction test was composed of 0.5 µL of each primer (20 pmol/ µL), 1.0 µL probe (6 pmol/ µL), and 4 µL of target RNA. Cycling conditions were 45 °C for 10 min, 95 °C for 10 min, 40 cycles of 94 °C 10 sec, 57 °C for 30 sec (optics on), and 72 °C for 10 sec. Comparisons were performed of the original test, tests with either primer or probe replaced, or comparisons with both the primer and probe replaced using the AgPath-ID reagents. The optimized test was used to amplify the RNA extracted from the Mexican FTA card samples that contained aMPV-A-specific NGS reads, or reference aMPV subtypes A, B and C isolates available at SEPRL.

## 3. Results

### 3.1. NGS-Based Nontargeted Virus Discovery

Although FTA cards produce lower quantities of partially degraded total RNAs compared to conventional methods of sample collection and processing, our protocols produced high numbers of total trimmed/filtered NGS reads (*n* = ~128,000–831,000 range) and low proportions of host-specific reads (*n*= ~1.8–53% range) compared to those typically obtained from field-collected samples depending on the sample type (tissue vs. respiratory) [48,62].

NGS detected aMPV RNA in 10.07% (*n* = 15; all from broiler chickens) of the analyzed 149 respiratory samples (Table 1). Note that one of the 15 samples (choana/lung sample 2723/21) is not included in Table 1; the sample contained only 509 aMPV-specific reads that were assembled to a consensus sequence of partial M2-gene (804 in length), but it was not further analyzed in the current study. In 10 out of the 15 samples, RNAs were detected of other viruses of families *Reoviridae* (avian orthoreovirus; AvRV; sample 2723/21), *Astroviridae* (avian nephritis virus; ANV; *n* = 1 sample), *Orthomyxoviridae* (influenza A virus subtype H5N2; *n* = 3 samples), *Coronaviridae* (infectious bronchitis virus; IBV; *n* = 7 samples, including sample 2723/21), *Paramyxoviridae* (Newcastle disease virus; NDV; *n* = 2 samples), and *Picornaviridae* (sicinivirus; SiV; *n* = 4 samples). Five of the 10 samples contained multiple (*n* = 2–4) coinfecting viral agents, while eight contained bacterial species that are pathogenic to or are associated with diseases in poultry, i.e., *Ornithobacterium rhinotracheale*, *Bordetella avium*, *Enterococcus* species, *Salmonella enterica*, and *Streptococcus pluranimalium* (Table 1).

None of the analyzed 114 immunological or the two digestive tissue samples contained detectable aMPV RNAs. However, RNAs of other avian respiratory viruses were detected in the immunological samples, including IBV (*n* = 37 samples), infectious bursal disease virus (IBDV; *n* = 24 samples), NDV (*n* = 13 samples), and H5N2 (*n* = 3 samples). Further, IBV (in all 37 immunological samples) was coinfected with one or more of the three respiratory viruses. This differed from the respiratory samples, where IBV was the most common respiratory virus in the respiratory samples (detected in 47.65%; *n* = 71 out of the 149 respiratory samples), its coinfection with other respiratory viruses was in 17 samples only, and mostly associated with NDV and/or aMPV (*n* = 9 and 6 samples, respectively).

### 3.2. Sequence Assembly

Seven full genome sequences of aMPVs were de novo assembled (13,288–13,381 nt in length) with the median depth ranging from 308X to 1562X (Table 1). The assembled genome sequences are consistent with the organization and sequence lengths of previously reported aMPVs [6]. The full genome sequences have been deposited in GenBank under accession numbers ON854003, ON854004, ON854006, ON854007, ON854012, ON854013, and ON854014. In addition to the full genome sequences, another set of seven samples contained aMPV-specific reads (*n* = 165–8107) that were reference-based assembled to complete CDS of the viral genes N (*n* = 2 samples), P and M (*n* = 7 samples each), F and L (*n* = 1 sample each), M2 (*n* = 6 samples), SH (*n* = 5 samples), and G (*n* = 2 samples). The sequences of the individual CDS have been deposited in GenBank under accession numbers OP359606 to OP359630.

### 3.3. Sequence Analysis

**Figure 1:**
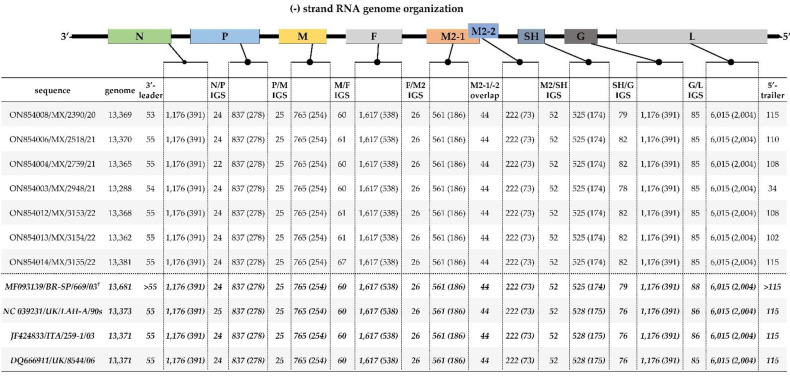
Genome organization and sequence lengths (nt and deduced amino acid (aa) residues (in brackets; excluding the final stop codon)) of the CDS and non-coding intergenic sequences (IGS) between adjacent genes of the seven complete genome sequences of aMPVs identified in the current study compared to previously reported Eurasian and Brazilian aMPV-A strains (four sequences shown at the bottom of the Figure in italicized bold font). Isolate/strain names include GenBank accession number, avian species, country, and year of sample collection. † strain BR-SP/669 has extended 3′-leader (*n* = 186 nt) and 5′-trailer (*n* = 291) regions.

All seven full genome sequences contain the eight known aMPV genes flanked by 3′-leader and 5′-trailer regions with a 3′-leader-N-P-M-F-(M2 [M2-1/M2-2])-SH-G-L-trailer-5′ genomic organization consistent with aMPVs [13]. Both the organization and nt lengths of the assembled consensus genome sequences of the seven Mexican isolates are comparable to previously reported European and Brazilian aMPV-A strains (Figure 1). Similar results were obtained from all the CDS of the individual genes obtained from the reference-based assembly, except in the sequence 2582/21 (GenBank accession number OP359618), which lacked reads coverage on 11 bases at the 3′-end of the M2-1 gene (but the full-length CDS has complete read coverage).

#### 3.3.1. Non-Coding Intergenic Sequence (IGS) and Extragenic (3′-leader/5′-trailer) Genomic Regions

As shown in Figure 1, the numbers of nt in the IGS of the seven aMPVs was consistent with other aMPVs between genes P/M (*n* = 25 nt), F/M2 (*n* = 26 nt), M2-1/M2-2 overlap (*n* = 44 nt) and M2/SH (*n* = 52 nt). The lengths of four IGS (between genes N/P, M/F, SH/G, and G/L) of the Mexican isolates slightly differ from the European and Brazilian aMPV strains (Figure 1). Similar to the European and Brazilian aMPV strains, the 3′-leader of the Mexican sequences is 55 nt-long, except in sequences 2390/20 and 2948/21 (GenBank accession numbers ON854008 and ON854003, respectively), which have ambiguities of the reads at the first two nt at the N-terminus. The lengths of the 5′-trailer genomic regions of two of the seven Mexican sequences are 115 nt-long, similar to the Eurasian aMPV strains, but vary in the other six sequences due to lack of reads coverage or ambiguities.

**Figure 2:**
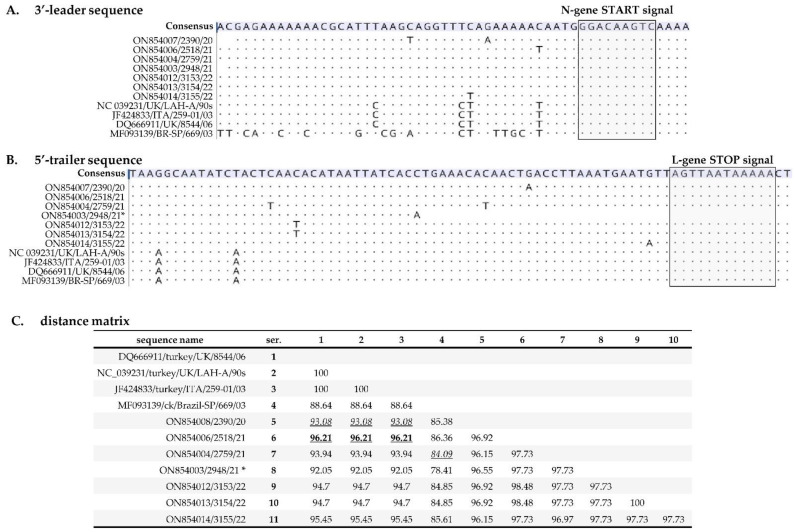
Analysis of (**A**) the 3′-leader and (**B**) the 5′-trailer regions of the aMPVs identified in this study compared to previously reported European and Brazilian strains. The transcriptional start and stop signal sequences are boxed for N- and L-genes, respectively. Dots in the alignments indicate identical nt. (**C**) Distance matrices of concatenated sequences of the 3′-leader and 5′-trailer regions. The highest and lowest nt identities between the Mexican, European, and Brazilian strains are underlined (in bold and italicized fonts, respectively). The low nt identities in the sequence 2948/21 (marked with asterisk “*”) could be attributed to the incomplete 3′-leader (n = 54 nt) and 5′-trailer (n = 34) as explained in the text. Isolate/strain names include GenBank accession number, avian species, country, and year of sample collection.

Isolated nt variations are present in the 3′-leader and 5′-trailer regions of the Mexican isolates compared to other aMPV-A strains (Figure 2A,B), but the transcriptional gene start signal (GGGACAAGT) and stop signal (AGTTA(Xn)polyA) sequences are conserved, a typical feature of pneumoviruses [17,63]. Compared to the European and Brazilian strains, the nt identities of the concatenated sequences of the 3′-leader and 5′-trailer regions of the Mexican aMPVs range from 92.05% to 96.21%, respectively (Figure 2C).

#### 3.3.2. Coding Sequence (CDS) Regions

All the CDS have the same lengths across all the Mexican aMPVs identified in this study: N (*n* = 1176 nt; 391 aa), P (*n* = 837 nt; 278 aa), M (*n* = 765 nt; 254 aa), F (*n* = 1617 nt; 538 aa), M2-1 (*n* = 561 nt; 186 aa), M2-2 (*n* = 222 nt; 73 aa), SH (*n* = 525 nt; 174 aa), G (*n* = 1176 nt; 391 aa) and L (*n* = 6015 nt; 2004 aa), which are consistent with European and Brazilian strains used in our analyses (Figure 1).

### 3.4. Genetic Relationships of the Mexican and Other aMPVs

As summarized in Table 2, the full genome sequences of the Mexican aMPV isolates, when compared with published genomes, have the highest nt identities to European aMPV-A strains UK/8544/06 (97.22–97.47%), ITA/259-01/03 (97.14–97.39%) and UK/LAH-A/90s (97.08–97.33%) and lowest identities to the Brazilian strain BR-SP/669/03 (96.96–97.22%). The G-gene sequences of the Mexican isolates are most similar to strain UK/8544/06 at both the nt (95.07–95.83%) and aa (90.28–92.84%) levels, and least similar to strain UK/CVL14-1/88 (94.30–95.24% and 89.26–91.82% at the nt and aa levels, respectively). Comparative homologies of all eight viral genes between the Mexican isolates and European and Brazilian aMPV-A strains are presented in Table S1.

#### 3.4.1. Viral Membrane Proteins G, F, and SH

**Figure 3:**
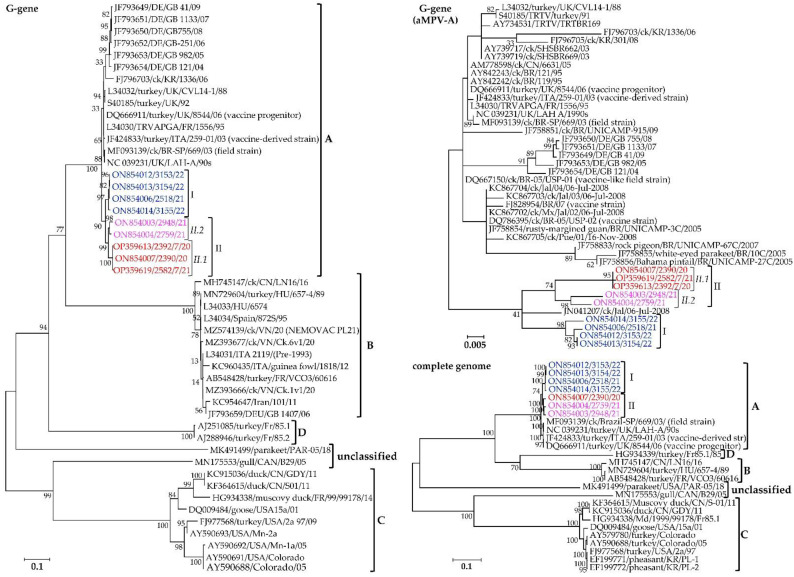
Relationships between the Mexican aMPV-A isolates identified in the current study (highlighted in color) and previously reported aMPVs based on the attachment (G) gene and the full-length genome nt sequences. Isolate/strain names include GenBank accession number, avian species, country, and year of sample collection.

The G, F, and SH genes encode the surface glycoproteins of aMPVs, with the G- and F-proteins forming the viral membrane surface projections. Based on G-gene nt sequences, the Mexican isolates identified here form two distinct clusters (clusters I, II.1, and II.2), separate from previously reported Eurasian (UK, Italy, Germany, China, and South Korea) and South/Latin American (Mexico and Brazil; including Brazilian vaccine-like strains) aMPV-A strains (Figure 3). The complete genome sequences of the Mexican clusters I and II isolates have within-group mean p-distances of 0.0045 and 0.0147 and overall nt similarities of 99.6% and 99.8%, respectively. For the G-gene, clusters I, II.1, and II.2 sequences have within-group mean p-distances of 0.0058, 0.0158, and 0.0006, with overall nt similarities of 99.4%, 98.5%, and 99.9%, respectively. Based on the full CDS of the G-gene, the overall similarities between the aMPV-A strains available in GenBank and the Mexican isolates is 95.9%, with the Mexican isolates and the other aMPV-A strains having within-group mean p-distances of 0.0292 and 0.0229, respectively.

**Figure 4:**
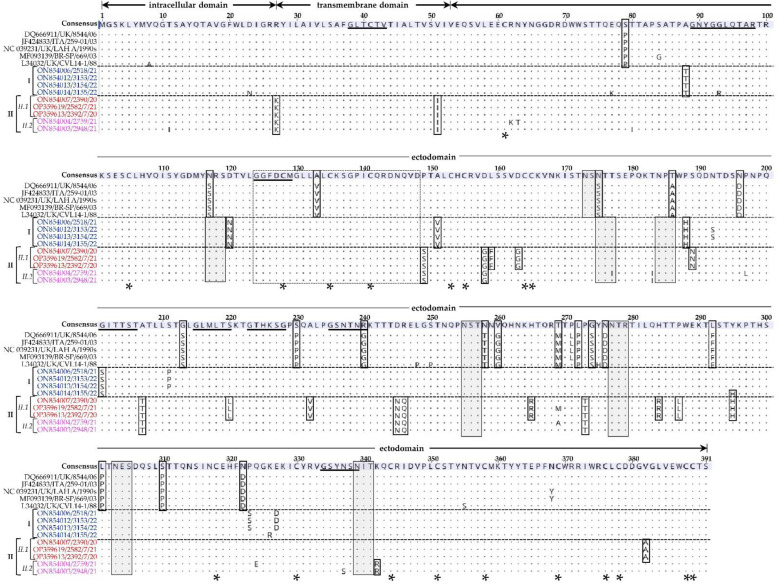
Analysis of aa variations and domains of the G-protein sequences of the Mexican aMPV-A isolates identified in the current study compared to other aMPV-A strains. The horizontal dotted lines group the Mexican sequences (highlighted in color) based on the phylogenetic clusters I, II.1, and II.2 (see Figure 3). The dots in the aligned sequences indicate identical aa residues. The horizontal double-headed arrows above the consensus sequence indicate the intracellular, transmembrane, and extracellular domains. Open boxes in the alignment indicate aa variations between the aligned sequences. Asterisks indicate conserved cysteine residues (*n* = 19). A stretch of 25 aa residues (positions 124–148; highlighted in dotted open box) in the ectodomain is conserved in aMPV-A and B strains [2].Asterisks (*) indicate conserved cysteine residues (n = 19). Predicted N-linked glycosylation sites are highlighted in shaded boxes. N-myristylation sites are underlined in the consensus sequence. Isolate/strain names include the GenBank accession number, avian species, country code, and year of sample collection.

Domain organization of the G-protein of the Mexican aMPVs is consistent with type II membrane proteins, consisting of an intracellular domain (aa residues 1–27), a transmembrane domain (aa residues 28–53), and an ectodomain (residues 54–391); all the sequences have 19 conserved cysteine residues (Figure 4), which is consistent with other previously reported aMPV-A strains [2]. Multiple alignments of the deduced that the aa sequence of the G-proteins concurred with the phylogenetic clustering of the Mexican isolates, with 38 aa variations amongst clusters I, II.1, and II.2, and when compared to strains LAH-A/90s, BR-SP/669/03 and 8544/06 (Figure 4). One of the aa variations occurs in a conserved stretch of 25-aa residues within the ectodomain (residues 124–148 as reported in other aMPV-A and -B viruses [2]) where the Mexican aMPVs have adenine at position 133 (A133) compared to V133 in other aMPV-A viruses. The A133 is present in the G-protein sequences of aMPV-B viruses such as the French strain AJ251085/turkey/Fr/85.1 and the Italian strain L34031/TRVAPGB/ITA-2119 [2]. Another difference is the presence of seven potential *N*-linked glycosylation sites in the Mexican aMPVs compared to five sites in other aMPV-A strains.

**Figure 5:**
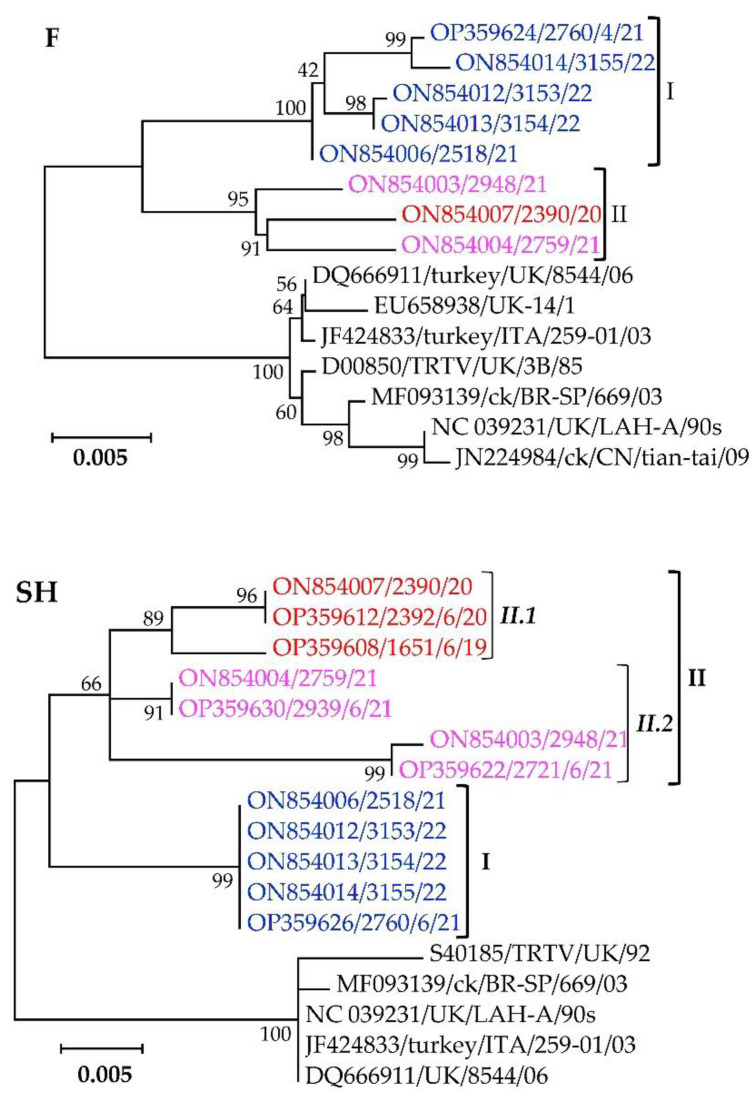
Phylogenetic analysis of the fusion (F) and small hydrophobic (SH) glycoproteins of the Mexican aMPV-A isolates (highlighted in color) identified in this study and other previously reported aMPV-A strains. Isolate/strain names include GenBank accession number, avian species, country, and year of sample collection.

The second component of the pneumovirus membrane is the highly antigenic but conserved F-glycoprotein. Phylogenetic clustering of the F-gene nt sequences separates the Mexican cluster I and II isolates in a distinct clade from other aMPV-A strains, all supported by high bootstrap values (Figure 5). The Mexican cluster I and II isolates have within-group mean *p*-values of 0.0056 and 0.0116, with overall nt similarities of 99.4% and 99.8%, respectively.

Results obtained from the analyses of the deduced aa residues of the F-protein are shown in Figure S1. The F-protein is conserved across the aMPV-As, including the arginine-rich cleavage site motif (^99^ R-R-R-R ^102^), cysteine residues (*n* = 16), the atypical integrin-binding domain (position ^329^ R-D-D ^331^, which is critical for the fusion activities), and the N-terminal signal peptide sequence (position aa 1–18) of subunit F_2_ (except at position G16) in the Mexican aMPVs compared to S16 in all the other aMPV-A strains used in the analyses (Figure S1). In addition to position G16, there are several other variations both amongst the Mexican isolates and compared to other aMPV-A strains. Four of the variations (S54N, K233R, S345L, and F514S) distinguish the Mexican isolates from all seven Eurasian/Brazilian aMPV-A strains used in the analyses. Seven other variations distinguish the Mexican clusters I and II from each other and other previously published aMPV-A strains. Three of these (K94R, G264D, and K293R) are unique to the Mexican cluster II isolates compared to the Mexican cluster I isolates and the other aMPV-A strains. The other four of the seven variations (R179Q, E294K, G387S, and V506L) distinguish the Mexican cluster I isolates from cluster II and other aMPV-A strains. Some of these variations are notable in the aMPV-As. For instance, a mutation that resulted in a K294E change enhanced the protective capacity of an attenuated UK/8544/06 strain [64], while variations at positions 233, 264, 293–294, 345, and 387 are in two regions in the F_1_ ectodomain, section 4 (aa residues 211–310) and section 5 (aa residues 336–479) that have been associated with the specific binding of neutralizing antibodies during immune responses to aMPV-A and B infection in birds (but not in the distantly related subtype C viruses) [65].

The segregation of the Mexican aMPV-A cluster I, II.1, and II.2 isolates from each other, and other aMPV-A strains based on the SH-glycoprotein is apparent from the topology of the phylogenetic tree shown in Figure 5. The SH glycoproteins are relatively conserved across the analyzed viruses, with the Mexican cluster I isolates being 100% identical, while cluster II.1 and II.2 isolates have overall nt identities of 99.2% and 98.5% with within-group mean *p*-values of 0.0076 and 0.149, respectively. Analyses of the deduced aa residues of the SH glycoprotein (Figure S2) confirm the conservation observed in the phylogenetic tree, including the 14 cysteine residues reportedly conserved in aMPV subtypes A, B and D [66]. Eight aa variations in the SH glycoprotein distinguish the Mexican clusters amongst themselves and from the other aMPV-A strains (Figure S2). Two of the variations (K16T and L106P) distinguish the Mexican isolates from all the other aMPV-A strains used in the analysis. Three other variations (I62V, G102R, and I174N) differentiate the Mexican cluster I isolates from all other aMPV-A stains used in the analysis, including the Mexican cluster II.1 and II.2 isolates. Only two variations (R14K and K155E) distinguish the Mexican cluster II.2 isolates from other aMPV-A viruses, while T86M distinguishes the Mexican cluster II isolates from other aMPV-A viruses.

#### 3.4.2. Viral Ribonucleoprotein Complex Proteins N, P, and L

**Figure 6:**
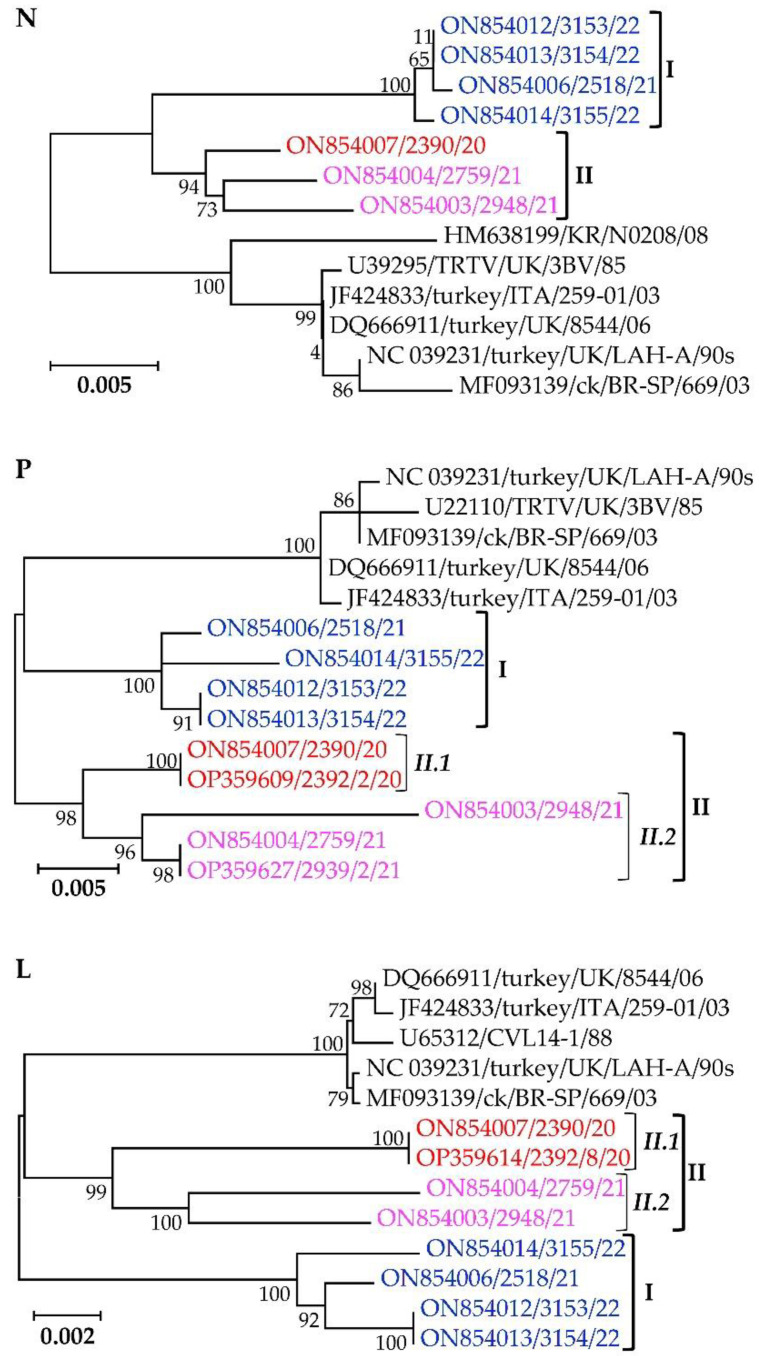
Phylogenetic trees of the components of pneumovirus ribonucleoprotein complex (nucleocapsid (N), phosphoprotein (P), and large polymerase (L) proteins) of the Mexican aMPV-A isolates identified in the current study and Eurasian and Brazilian aMPV-A strains. Isolate/strain names include GenBank accession number, avian species, country, and year of sample collection.

Similar to the membrane G and SH proteins, the sequences of the pneumovirus ribonucleoprotein complex components (i.e., N, P, and L proteins) group the Mexican cluster I and II isolates separate from other aMPV-A strains, with the phylogenetic trees supported by high bootstrap values (Figure 6). The N-gene nt sequences of the Mexican clusters I and II isolates have overall nt similarities of 99.9% and 99.0% with within-group mean *p*-values of 0.0013 and 0.0096, respectively. Clusters I, II.1, and II.2 isolates have P-gene overall similarities of 99.4%, 100%, and 98.7% and within-group mean *p*-values of 0.0064, 0.0 and 0.0127, respectively, and for the L-gene, overall nt similarities of 99.5%, 99.9% and 98.5% and within-group mean *p*-values of 0.0006, 0.00, and 0.0016, respectively.

The N, P, and L proteins are highly conserved, with only five and eight aa variations distinguishing the N and P proteins of the Mexican isolates from other aMPV-A strains (Figure S3). Although none of the five aa variations in the N protein are located within the conserved regions [67], all of them distinguish the Mexican isolates from other aMPV-A strains (T45I, S101P, S105P, T129A, and R356K).

Three of the eight aa variations in the P-protein distinguish the Mexican isolates from the other aMPV-A strains (P73S, E265D, and D267N), two distinguish the Mexican cluster II isolates from all other aMPV-A strains (A90V and R262G), another two sets apart the Mexican cluster II.2 isolates from other aMPV-A strains (S/G212D and H267N) and one variation unique to Mexican cluster II.1 isolates (R84K). Other isolated variations in individual sequences across the N and P sequences are shown in Figure S3.

Multiple alignments of the L-protein sequences revealed high conservation across the aMPVs used in the analysis, including four of the functional domains of the protein [66,68,69], which are located at positions 631–643 (motif A), 702–727 (motif B), 741–750 (motif C), and 808–820 (motif D). However, there are 19 amino acid variations when comparing the Mexican isolates and previously reported aMPV-A strains, which can be grouped into five categories as summarized in Table 3.

The first category consists of eight positions (Y44H, R313Q, A817S, V862L, D877N, L1103M, H1354R, and T1557N) where the Mexican isolates vary from the other aMPV-A strains. The second category consists of five variations (C133R, S328G, V1204A, L1397P, and G1739S) that are unique to the Mexican cluster I isolates, while the third and fourth categories are unique to the Mexican cluster II.1 (S71R) and cluster II.2 (L1791V and R1912K) isolates. The last category has three positions (S4P, T1606L, and L1690V) that are common to both the Mexican cluster I isolates, and the previously reported aMPV-A strains compared to the Mexican cluster II isolates. Only 1 of the 19 variations (A817S) is found in 1 of the 4 pneumovirus-conserved L-protein motifs (motif D found within domain III [66,69]; motifs A-C are 100% conserved across the analyzed aMPV-A viruses.

#### 3.4.3. Viral Replication and Assembly Matrix Glycoproteins M and M2

**Figure 7:**
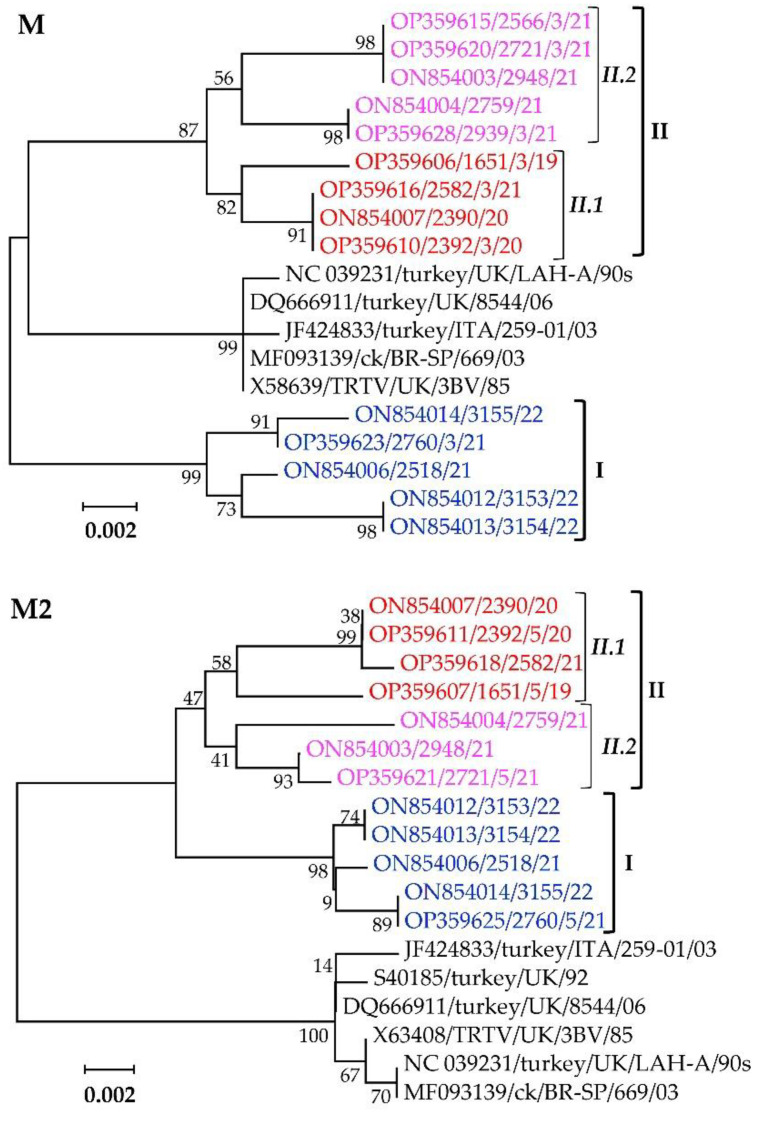
Phylogenetic trees of the matrix (M) and accessory matrix (M2) glycoproteins of the Mexican aMPV-A isolates and aMPV-A strains from the UK and Brazil. Isolate/strain names include GenBank accession number, avian species, country, and year of sample collection.

Phylogenetic analyses of the M and M2 glycoproteins separate the Mexican clusters I, II.1, and II.2 isolates from other aMPV-A strains (Figure 7). The M proteins of the Mexican clusters I, II.1, and II.2 isolates have overall similarities of 99.3%, 99.7%, and 99.5% and within-group mean *p*-values of 0.0071, 0.0033, and 0.0055, respectively, while the M2 proteins have overall similarities of 99.7%, 99.4%, and 99.3% and within-group mean *p*-values of 0.0028, 0.0058, and 0.0052, respectively.

Analyses of the deduced aa residues of the M protein showed high conservation across all of the sequences, with only V220M variation between the Mexican cluster II and all other analyzed aMPV-A strains (data not shown). For the accessory M2 glycoprotein, the Mexican isolates differ from the other aMPV-A strains at three positions in the M2-1 protein (V47L, N142S, and S169P) and two positions in the M2-2 protein (K8R and I46V). However, the M2-2 protein of strains UK/LAH-A/90s, UK/3BV/85 and BR-SP/669/03 differ from all other analyzed aMPV-A strains at position K26R.

### 3.5. Revised aMPV-A rRT-PCR Test

A previously published AmPV rRT-PCR test [10] was evaluated that showed high sequence similarity of both forward and reverse primers and probes with average single nucleotide polymorphism (SNP) scores of 4.3 to 9.6 for the entire primer or probe [10]. Although the published primers were well conserved, the reverse primer had high heterogeneity at the 3′ -2 nt position, which might reduce sensitivity for mismatched isolates. Using the alignment with the SNP data, an alternative reverse primer and probe were selected that had even higher sequence similarity that lowered the SNP average from 9.6 to 3.5 for the reverse primer and negated the heterogeneity issue at the 3′ end of the primer, and a change in the probe reduced the SNP average from 4.4 to 1.4.

Based on the C*_T_* values of the 10-fold diluted RNAs, similar results were obtained from the alternative primers/probes set of this study and the set described in the published set, when used together or separately. There was negligible difference in the results obtained from the three-step cycling conditions that were used in the current study compared to the rRT-PCR test conditions commonly used in the USA. However, the replacement of both the reverse primer and probe with the new cycling conditions showed slightly lower C*_T_* values compared to the original test. Based on these data, the revised test was used for confirmatory testing of a selection of the Mexican FTA samples that contained aMPV-specific NGS reads and compared with representative AmPV-B and C viruses. All the aMPV-A-positive samples by NGS were positive on the revised aMPV rRT-PCR test. One of the aMPV-B viruses also tested positive on the AmPV-A test, but the aMPV-B test gave a C*_T_* value 10 cycles lower than the aMPV-A test. This result is not surprising because results from the published aMPV-A test showed that most samples with high concentrations of aMPV-B would be positive [10]. None of the analyzed aMPV-C viruses tested rRT-PCR-positive.

## 4. Discussion

A high prevalence of aMPV-A and B has been reported in high-density poultry farms (chicken and turkey flocks) and free-living wild birds in Brazil [45,46,70]. In Mexico, circulation of aMPV-A was highlighted in Jalisco and Puebla in 2007–2008 from samples collected from non-vaccinated chicken flocks, which, prior to the time of sampling, had overt clinical signs consistent with respiratory infections [44]. However, with the exception of one full genome sequence of the Brazilian aMPV-A virus, the Mexican and Brazilian aMPV-A sequences available in GenBank are partial gene sequences (mostly G-gene fragments of ~ 200 nt in length) produced using RT-PCR-based amplification and sequencing. Since the 2007–2008 detection of aMPVs, no additional detection of the virus or attempts to characterize the subtypes circulating in Mexican poultry have been reported.

A key finding of the current study is that, despite sequencing relatively high numbers of immunological samples (*n* = 114; compared to 149 respiratory samples), aMPV RNA was detectable only in the respiratory (choana/lung) samples. We also found instances of coinfection of aMPVs with respiratory viruses (i.e., IBV, H5N2, and NDV) and pathogenic bacterial species in a majority of the aMPV-positive samples, but this is not unexpected because coinfections are often observed in chickens [71]. The most common coinfecting viral and bacterial agents in the current study were IBV and gram-positive coccus bacterial species (*E. cecorum* and *S. pluranimalium*), which were detected in seven and six aMPV-positive samples, respectively. Coinfections with multiple viruses are postulated to enhance pathogenicity, complicate the diagnosis of the virus and increase viral shedding [72,73].

Globally, only four aMPV-A full genome sequences are available in public genomic databases, three of which were detected in turkeys from Europe (strains UK/LAH-A/90s, UK/8544/06, and ITA/259-1/03), and one detected from a chicken in Brazil in 2003 (BR-SP/669/03). With few exceptions, such as the lengths of the IGS between some of the viral genes, there were no structural variations in the genome organization and the lengths of the gene CDS of the Mexican aMPV-A isolates reported in the current study, and when compared to the Eurasian and Brazilian aMPV-A strains. In addition to being the first report of genome sequence variations of Mexican aMPV-A viruses, our datasets are valuable for future investigations on the molecular epidemiology of the viruses. More importantly, since the Mexican aMPV-A isolates are phylogenetically distinct from the previously reported vaccine and vaccine-like field strains (overall nt similarity of 95.9% based on the nt sequences of the complete CDS of the G-gene), our data should inform on vaccine development in Mexico and the region. This is because genetic differences between endemic field and commercial vaccine strains could compromise vaccine efficacy and eventually influence the emergence of novel variants in the event of reversion to virulence when live vaccines are used [23,74,75].

It is notable that the Mexican aMPV-A viruses identified here group into distinct clusters separate from Eurasian and Brazilian viruses based on alignments of the complete genome and individual gene sequences, including the G-gene that is used in pneumovirus classification. Only one of five partial G-gene sequences was reported from Jalisco in the 2008 (JN041207/ck/MX/Jal-06/08; 204 nt in length) group with the Mexican isolates identified in the current study. The presence of numerous variations in the CDS of the three surface glycoproteins (G, F, and SH) and other genes (e.g., L and P) amongst the Mexican isolates, when compared with the other aMPV-A viruses, is not totally unexpected, keeping in mind that in addition to the error-prone viral RdRp-mediated genome replication, aMPVs have negative-sense genomes, which are highly susceptible to mutations [76]. Currently, the public databases have over a decade-long scarcity of full genome and G-gene sequence data of aMPV-A viruses from Latin/South America and across the globe, which limits further comparative sequence analysis of the Mexican isolates in the current study to understand the source and evolutionary histories of these viruses. Although it remains to be experimentally demonstrated whether the genetic variations observed in the Mexican isolates (compared to other aMPV-A strains) have any effects on their pathobiology, the phylogenetic clustering and the aa sequence variations could indicate an emergence and circulation of a unique group of aMPV-As in Mexico. With the role of wild birds in the spread of aMPVs remaining undetermined, it cannot be ruled out that these viruses could spread from and to other countries. Since the results from the current study heighten the initial 2014 recognition of circulation of aMPV-A in Jalisco and Puebla states of Mexico, it is imperative to have available validated diagnostic tests for sensitive and specific testing of aMPV-A viruses in chickens and other poultry species.

Accurate detection is a critical prerequisite in the effective management and control of microbial agents that cause respiratory diseases. Mostly because of its sensitivity and rapid turnover time, RT-PCR has generally been accepted as the gold standard for the detection and subtyping of multiple avian viral pathogens. However, this and other conventional molecular assays target the amplification of specific viral genes, thus limiting the genetic information on the viral agents and, in the case of avian RNA viruses that have high mutation rates, the methods must be frequently optimized and validated for sensitivity and specificity. The nontargeted high-throughput NGS approach that we utilized in this study demonstrates its potential for broad-spectrum pathogen screening while simultaneously providing highly specific genetic information on the identified pathogens. Arguably, the perceived loss of sensitivity of the nontargeted approach in the detection of viral pathogens remains to be addressed to make it as sensitive as current molecular diagnostic approaches (e.g., qRT-PCR). The higher cost, longer processing time, and bioinformatics analysis requirements have also limited the broad acceptance of NGS technology in diagnostics, but the richness of information provided together with the improved methodology raises the expectation that NGS will become a viable alternative for routine active surveillance in the future.

In the USA, aMPV-A would be considered a foreign animal disease, and early detection could help reduce the spread and potentially eliminate the virus. Although several traditional RT-PCR tests that detect PCR bands on agarose gels had been previously described, these types of tests are not often used in diagnostic laboratories because of the additional labor costs and the greater potential for cross-contamination. In the USA, real-time PCR and RT-PCR tests are generally preferred. Two rRT-PCR diagnostic tests have been described for aMPV-A, one for the N gene and the other for the G gene [10,11]. For the current study, the G gene-based test was selected for further evaluation because of the greater extent of testing that was reported in the original paper [10] for this gene, as well as the greater availability of sequence data that was available for the G gene as compared to the N gene. Using SNP analysis, each nt position of the primers and probes was evaluated. In general, the primers and probes were highly conserved, but the same SNP analysis suggested that sliding the probe and the reverse primer a few nt forward or backward could improve the sequence conservation. The use of SNP analysis has been described previously to develop new rRT-PCR tests for ND [77].

Comparison testing between the original aMPV-A test and the revised test showed comparable results. The other major change in the revised test was to change the cycling conditions and amplification reagents to be more compatible with what is used in many veterinary virology laboratories in the USA. The cycling conditions in particular use a three-step cycling program with an annealing temperature of 57 °C, and these cycling parameters are also used for other poultry pathogens in the USA, including influenza A viruses. Having the same cycling conditions for multiple tests allows for the testing of multiple pathogens on the same 96-well PCR plate. Similarly, porting the aMPV-A test to AgPath RT-PCR reagents is advantageous to U.S. laboratories to avoid having to stock multiple amplification reagents in the laboratory, particularly for tests that may only rarely be used. The bioinformatics analysis of the sequenced viruses and testing of representative Mexican aMPV-A positive field samples showed sensitive amplification and reasonable assurance that these tests can be used for the detection of aMPV-A viruses. The only concern is that the revised aMPV-A rRT-PCR would also detect some aMPV-B viruses if they were at a high concentration in particular samples, an issue that was also highlighted in the original G-gene rRT-PCR test manuscript [10].

## 5. Conclusions

Our study reports for the first time the full genome sequences of seven aMPV-A isolates in respiratory samples of commercial broiler chickens from Mexico. Additionally, we report the complete CDS of various aMPV-A genes identified in another seven respiratory samples. Based on our sequence analyses at both the nt and aa levels, the Mexican aMPV-A isolates identified in the current study cluster separately from all previously published Eurasian, and Brazil aMPV-A strains and vary amongst themselves as demonstrated by partitioning into clusters I and II, with cluster II further segregating into two distinct subclusters. Since the viruses were identified in samples collected from commercial farms, future studies should consider surveillance of backyard poultry. Furthermore, our data points to a future need to look into the question of the potential roles that wild birds may play in the epidemiology and the spread of aMPVs in commercial poultry. Our data are valuable in the review and further development of vaccines based on the field strains that are circulating in Mexico because the use of vaccine strains that genetically differ from field strains can affect vaccine efficacy and contribute to the emergence of new variants. Finally, we used our data in combination with sequence data from other pneumoviruses available from GenBank to revise a previously published rRT-PCR test, which resulted in improved cycling and amplification conditions compatible with a diagnosis of aMPV-A viruses, multiple other poultry viruses, and improved sensitivity to aMPV-A.

## Figures and Tables

**Table 1:** Summary of the assembly of 14 aMPV sequences and other coinfecting microbial (viral and bacterial) agents detected in clinical samples collected from commercial broiler chickens in the current study. aMPV RNA was detected by NGS in 10.07% of the analyzed 149 respiratory (choana/lung) samples. Where applicable, viral and bacterial agents found to coinfect with aMPVs are shown. Median depth coverage of full genome sequences is highlighted (bold underlined font).

Sample ID ^a^	Sampled Tissue	Sampling Date	No. of Filtered/Trimmed NGS Reads			No. of aMPV Contigs ^c^	Sequence Coverage Depth ^d^	Genomic Region (Consensus seq Length; no. of Bases)	Other Agents Identified (NGS Reads) ^e^	
			Total	Chicken	aMPV ^b^				Viruses	Bacteria
1651/19	choana	1-May-19	831,098	49.9%	4194	N/A	N/A	full CDS; P (852), M (837), M2 (787), SH (525)	IBV (809)	
2390/20	Choana; lung	9-December-20	279,560	6.7%	30,237	4	1|98|**308**|933|2794	full genome (13,369)	IBV GI-3 (341)	*B. avium* (22,902); ORT (19,718); *S. pluranimalium* (676)
2392/20	Choana; lung	9-December-20	438,683	10.5%	8107	N/A	N/A	full CDS; N (1176), P (852), M (837), M2 (787), SH (525), G (1176), L (6033)	IBV (87)	ORT (5118); *B. avium* (3374); *S. pluranimalium* (2269)
2518/21	Choana; lung	21-January-21	350,857	18.24%	44,220	1	1|190|**464**|920|12450	full genome (13,370)		
2566/21	Choana; lung	25-February-21	128,883	52.05%	165	N/A	N/A	full CDS; P (852), M (837)	IBV (620)	
2582/21	Choana; lung	27-March-21	162,856	32.68%	1985	N/A	N/A	full CDS; P (852), M (837), M2 (776), G (1176)		
2721/21	Choana; lung	6-June-21	600,788	31.38%	3463	N/A	N/A	full CDS; P (852), M (837), M2 (787), SH (525)	IBV (13,543); SiV (1126); ANV (182)	*E. cecorum* (2555); *S. pluranimalium* (32,279)
2759/21	Choana; lung	30-June-21	596,799	53.37%	106,434	2	0|770|**1430**|2658|10796	full genome (13,365)	SiV-A (4948); IBV GI-1 (262); AIV H5N2 (968); NDV II (306)	*S. enterica* (500); *E. cecorum* (1586)
2760/21	Choana; lung	30-June-21	476,815	49.64%	6426	N/A	N/A	full CDS; P (852), M (837), F (1617), M2 (787), SH (525)	SiV-A (2781); H5N2 (83.383)	*S. pluranimalium* (1342); *E. cecorum* (664)
2948/21	Choana; lung	17-November-21	386,419	31.27%	23,106	2	2|117|**277**|475|3522	full genome (13,288)	SiV-A (8550)	*S. pluranimalium* (7574); *E. cecorum* (1985)
2939/21	Choana	7-December-21	261,910	1.8%	1530	N/A	N/A	full CDS; N (1176), P (852), M (837), M2-2 (267), SH (525)		ORT (3495); *E. cecorum* (5990)
3153/22	Choana	27-April-22	232,341	20.23%	114,243	4	0|662|**1195**|2520|8314	full genome (13,368)		ORT (10,387); *B. avium* (720)
3154/22	Choana; lung	27-April-22	194,497	46.82%	31,306	1	1|181|**351**|708|2159	full genome (13,362)	H5N2 (2066); NDV V.5 (803)	*B. avium* (12,286)
3155/22	Choana	27-April-22	277,845	19.74%	141,073	1	1|913|**1562**|3067|12823	full genome (13,381)		*B. avium* (7022); *S. pluranimalium* (776)

^a^ Sample IDs include SEPRL code assigned to each sample during the MiSeq runs and the date of sample collection. ^b^ numbers of aMPV-specific reads used to recall the consensus sequence. ^c^ numbers represent the minimum|lower quartile|**median**|upper quartile|maximum) depth per position of the consensus sequences. N/A indicates “undetermined” ^d^ final genome consensus sequence lengths. N/A indicates “undetermined” ^e^ Abbreviations: aMPV, avian metapneumovirus; ANV, avian nephritis virus; *B. avium*, *Bordetella avium*; *E. cecorum*, *Enterococcus cecorum*; IAV, influenza A virus; IBV, infectious bronchitis virus; NDV, Newcastle disease virus; ORT, *Ornithobacterium rhinotracheale*; SiV, sicinivirus; *S. enterica*, *Salmonella enterica*; *S. pluranimalium*, *Streptococcus pluranimalium*.

**Table 2:** Comparison of nt and deduced aa (in brackets) identities between the full genome and G-gene sequences of the aMPVs identified in this study and their closest BLASTn matches. The highest and lowest identities are underlined (in bold and italicized fonts, respectively). Note: only the G-gene sequence of strain CVL14-1/88 is available in GenBank; the Mexican isolates 2392/20 and 2582/21 (marked with asterisk “*”) did not have full-length genome sequences. GenBank accession numbers are indicated in the isolate names.

Sequence	DQ666911/UK/8544/06		JF424833/ITA/259-1/03		NC_039231/UK/LAH-A/90s		MF093139 /BR-SP/669/03		L34032/UK/CVL14-1/88
	Genome	G-Gene	Genome	G-Gene	Genome	G-Gene	Genome	G-Gene	G-Gene
ON854007/2390/20	97.37%	*95.07% (90.28%)*	97.27%	*94.90% (90.28%)*	97.23%	*94.90% *(90.28%)	97.13%	*94.73% (89.77%)*	*94.30% (89.26%)*
OP359613/2392/20 *	N/A	*95.07% (90.28%)*	N/A	*94.90% (90.28%)*	N/A	*94.90% (90.03%)*	N/A	*94.73% (89.77%)*	*94.30% (89.26%)*
ON854006/2518/21	97.47%	95.83% (92.84%)	97.39%	95.83% (92.84%)	97.33%	95.66% (92.58%)	97.22%	95.49% (92.33%)	95.24% (91.82%)
OP359619/2582/21 *	N/A	95.15% (90.54%)	N/A	94.98% (90.54%)	N/A	94.98% (90.28%)	N/A	94.81% (90.03%)	94.39% (89.51%)
ON854004/2759/21	97.41%	95.49% (91.05%)	97.32%	95.32% (91.05%)	97.26%	95.32% (90.79%)	97.14%	95.15% (90.54%)	94.90% (90.03%)
ON854003/2948/21	97.37%	95.15% (91.56%)	97.29%	94.98% (91.56%)	97.21%	94.98% (91.30%)	97.10%	94.81% (91.05%)	94.73% (90.54%)
ON854012/3153/22	97.38%	95.83% (92.58%)	97.30%	95.66% (92.58%)	97.24%	95.66% (92.33%)	97.13%	95.49% (92.07%)	95.24% (91.56%)
ON854013/3154/22	97.39%	95.83% (92.58%)	97.31%	95.66% (92.58%)	97.25%	95.66% (92.33%)	97.13%	95.49% (92.07%)	95.24% (91.56%)
ON854014/3155/22	*97.22%*	95.66% (92.33%)	*97.14%*	95.49% (92.33%)	*97.08%*	95.49% (92.07%)	*96.96%*	95.32% (91.82%)	95.07% (91.30%)

**Table 3:** A summary of 19 aa variations found in the L-protein sequences of the Mexican aMPV-A isolates identified in this study compared to other aMPV-A viruses, which are grouped into five categories as described in the text. The corresponding nt codons are indicated in brackets next to the aa residues (shown in three-letter abbreviations). The variations that are unique to either the previously published aMPV-A strains or the Mexican clusters I, II.1, and II.2 isolates are highlighted in bold font type and underlined (refer to Figure 6 for the clustering of the Mexican sequences).

aa Position (from Start Met Residue)	aa Residue in Other aMPV-A Strains (UK/LAH-A/90s, UK/8544/06, ITA/259-10/03, and BR-SP/669/03)	aa Residue in Mexican aMPV-A Isolates		
		Cluster I	Cluster II.1	Cluster II.2
44	**Tyr (TAT)**	His (CAT)	His (CAT)	His (CAT)
313	**Arg (CGA)**	Gln (CAA)	Gln (CAA)	Gln (CAA)
817	**Ala (GCA)**	Ser (TCA)	Ser (TCA)	Ser (TCA)
862	**Val (GTT)**	Ile (ATT)	Ile (ATT)	Ile (ATT)
877	**Asp (GAT)**	Asn (AAT)	Asn (AAT)	Asn (AAT)
1103	**Ile (ATA)**	Met (ATG)	Met (ATG)	Met (ATG)
1354	**His (CAC)**	Arg (CGC)	Arg (CGC)	Arg (CGC)
1557	**Thr (ACT)**	Asn (AAT)	Asn (AAT)	Asn (AAT)
133	Arg (CGT)	**Cys (TGT)**	Arg (CGT)	Arg (CGT)
328	Gly (GGC)	**Ser (AGT)**	Gly (GGC)	Gly (GGC)
1204	Ala (GCC)	**Val (GTT)**	Ala (GCC)	Ala (GCC)
1397	Pro (CCA)	**Leu (CTA)**	Pro (CCA)	Pro (CCA)
1739	Ser (AGC)	**Gly (GGC)**	Ser (AGT)	Ser (AGC)
71	Arg (AGA)	Arg (AGA)	**Ser (AGC)**	Arg (AGA)
1791	Val (GTG)	Val (GTG)	Val (GTG)	**Ile (ATA)**
1912	Lys (AAG)	Lys (AAG)	Lys (AAG)	**Arg (AGG)**
4	Ser (TCC)	Ser (TCC)	Pro (CCC)	Pro (CCC)
1606	Thr (ACT)	Thr (ACT)	Ile (ATT)	Ile (ATT)
1690	Ile (ATT)	Ile (ATT)	Val (GTT)	Val (GTT)

## Data Availability

Genomic sequence data presented in this study have been deposited in GenBank under accession numbers: ON854003 to ON854016 (for whole genome sequences), and OP359606 to OP359630 (for other CDS sequences).

## Supplementary Materials

The following supporting information can be downloaded at: https://www.mdpi.com/article/10.3390/vetsci9100579/s1, Table S1: Comparative homologies of the full genome and gene sequences of the Mexican aMPV-A isolates identified in the current study and other previously reported aMPV-A strains. The highest and lowest identities are underlined (in bold and italics font, respectively). Isolate/strain names include GenBank accession number, avian species, country, sample ID, and year of sample collection. Figure S1. Comparative analysis of the fusion (F) glycoproteins of the Mexican aMPV-A isolates and other previously reported Eurasian and Brazilian aMPV-A strains. The horizontal dotted lines group the Mexican isolates into clusters I and II (see Figure 5). The horizontal double-headed arrows above the consensus sequence indicate F section 4 (aa residues 211–310) and section 5 (aa residues 336–479) of the F_1_ ectodomain that have been associated with modulating neutralizing immune responses in aMPV-A and B viruses [65]. Shaded and open boxes in the alignment indicate the main domains and aa variations of the proteins, respectively, while dots denote identical aa residues. Conserved cysteine (C) aa residues are indicated by asterisks (*). Isolate/strain names include GenBank accession number, avian species, country, and year of sample collection. Figure S2. Comparative sequence analysis of the small hydrophobic (SH) glycoprotein of the aMPVs identified in this study and three other aMPV-A viruses. Horizontal dotted lines group the Mexican sequences into clusters I, II.1, and II.2 (see Figure 5). Cytoplasmic domain and ectodomain are illustrated by horizontal double-headed arrows. Open boxes in the aa sequence alignment highlight aa variations between the sequences. Dots indicate identical aa residues. Asterisks (*) indicate conserved cysteine residues. Isolate/strain names include GenBank accession number, avian species, country, and year of sample collection. Figure S3. Comparison of the aa sequences of nucleocapsid protein (N) and phosphoprotein (P) of the Mexican aMPV-A isolates identified in the current study and other European and Brazilian aMPV-A strains. The horizontal dotted lines separate the Mexican clusters I, II.1, and II.2 isolates (see Figure 6). The double-headed horizontal arrows indicate functional domains homologous to the phosphoprotein of human pneumoviruses, while the shaded boxes indicate highly conserved regions in pneumoviruses [60,61,62]. Open boxes indicate amino acid variations amongst the viruses. Dots indicate identical aa residues. Isolate/strain names include GenBank accession number, avian species, country, and year of sample collection.
