# Supplementary figures and images for: Detection and Genome Sequence Analysis of Avian Metapneumovirus Subtype A Viruses Circulating in Commercial Chicken Flocks in Mexico
Source: Vet Sci. 2022 Oct 19;9(10):579. doi: 10.3390/vetsci9100579 (PMC9612082; doi:10.3390/vetsci9100579)

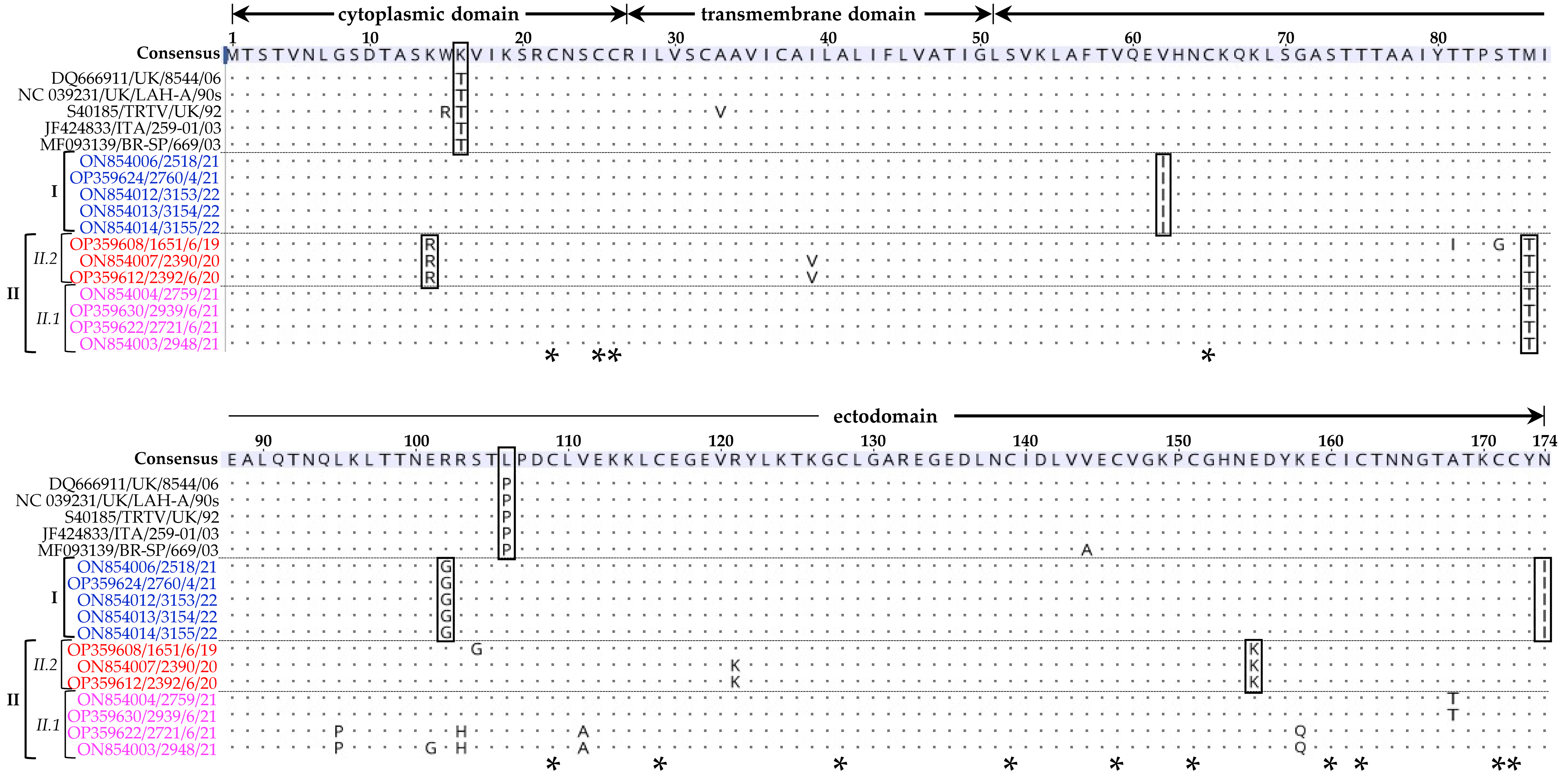

Supplement: Supplementary file 1 [file vetsci-09-00579-s001.zip › vetsci-1965045-supplementary/Supplementary Files/Figure S2.jpg]
